# Reducing spontaneous combustion propensity of lignite through functional group regulation by microbial flame retardant

**DOI:** 10.3389/fchem.2026.1781063

**Published:** 2026-02-18

**Authors:** Rile Ge, Liyuan Qin, Li Wang, Hui Song

**Affiliations:** 1 Engineering College, Hulunbuir University, Hulunbuir, China; 2 Hulunbuir College Mechanical Engineering Collaborative Innovation Research Center, Hulunbuir, China; 3 College of Mining, Chemistry and Chemical Engineering, Hulunbuir University, Hulunbuir, China

**Keywords:** functional groups, lignite spontaneous combustion, microbial flame retardant, *Pseudomonas putida*, spontaneous combustion propensity, thermogravimetric analysis

## Abstract

This study investigated the effects of microbial treatment on lignite functional group structure and thermal oxidation characteristics using *Pseudomonas putida* (CICC21884) through Fourier transform infrared spectroscopy (FTIR), thermogravimetry-differential scanning calorimetry (TG-DTG-DSC), spontaneous combustion propensity testing, and enzymatic activity assays. After 72 h of treatment, initial weight loss temperature increased from 140.99 °C to 162.12 °C (+21.13 °C), maximum weight loss rate decreased from 1.6%/min to 0.74%/min (−53.75%), and ignition temperature increased from 270.34 °C to 294.29 °C (+23.95 °C). Oxygen-containing functional groups decreased from 41% to 36%, aromatic groups increased from 17% to 30%, and hydroxyl content decreased from 39% to 31%. The spontaneous combustion propensity index peaked at 540.15 after 24 h (+28.71% vs. raw coal). Three extracellular enzymes—aromatic acid monooxygenase (22.0 U/mL), esterase (68.5 U/mL), and catalase (125.5 U/mg protein)—peaked at 24 h, showing temporal correlation with functional group modifications. Linear regression analysis across seven treatment conditions revealed general trends between functional group composition and thermal stability parameters, with weak to moderate correlations (R^2^ = 0.11–0.26) influenced by limited sample size and outlier effects. This study suggests that *P. putida* may degrade the oxygen-containing functional groups and promote coal aromatization, thereby inhibiting the low-temperature oxidation process of lignite and potentially providing an environmentally friendly biotechnological approach for lignite spontaneous combustion prevention.

## 1 Introduction

Lignite, as a low-rank coal, is highly susceptible to spontaneous combustion during mining, storage, transportation, and utilization may be related to its characteristics of high volatile matter, high oxygen content, and low fixed carbon content. This problem not only causes severe resource waste and economic losses but also triggers multiple environmental disasters including mine fires, surface subsidence, and atmospheric pollution (Kong et al., 2017; Yi et al., 2023). With the continuous development and utilization of low-rank coal resources, seeking efficient and environmentally friendly spontaneous combustion prevention technologies has become an urgent need for the sustainable development of the coal industry.

The spontaneous combustion of coal is essentially a low-temperature oxidation reaction between functional groups in coal and oxygen. Studies have shown that the types and contents of functional groups in coal macromolecular structures directly determine the oxidation activity and spontaneous combustion propensity of coal, with hydroxyl and carbonyl oxygen-containing functional groups becoming key factors in triggering coal spontaneous combustion may be related to their high chemical activity (Kong et al., 2017; Yi et al., 2023). During the low-temperature oxidation process of coal, oxygen-containing functional groups act as active centers, preferentially reacting with oxygen to generate peroxides and free radicals, initiating chain oxidation reactions, releasing heat, and accelerating coal temperature rise (Deng et al., 2025). As a low-rank coal, lignite has high oxygen-containing functional group content, low aromatization degree, and abundant aliphatic side chains in its macromolecular structure. These structural characteristics endow lignite with numerous active oxidation sites, enabling adsorption and chemical reactions with oxygen at ambient temperature. Therefore, the spontaneous combustion propensity of lignite is significantly higher than that of medium and high-rank coals (Gao et al., 2023; Zhang et al., 2022; Zhang Y. T. et al., 2021). Traditional coal spontaneous combustion prevention methods mainly include physical barrier methods and chemical inhibition methods. These methods have limitations such as complex equipment, short duration, easy re-ignition, poor adaptability to geological conditions, and potential for long-term harm to ecosystems (Zhao et al., 2023). Therefore, developing environmentally friendly new inhibition technologies has become a current research hotspot.

In recent years, biological prevention methods represented by microbial technology have gradually become a research hotspot in the field of coal spontaneous combustion prevention may be related to their environmental friendliness, cost-effectiveness, and sustainability (Deng et al., 2025; Wang et al., 2024; Zhao et al., 2023). Studies have shown that specific microbial strains can reduce the oxidation reactivity of coal by altering coal surface properties and degrading active functional groups through metabolic action (Dong et al., 2023; Koyunoğlu and Karaca, 2023). *Pseudomonas putida*, as an aerobic Gram-negative bacterium widely distributed in nature, possesses strong organic matter degradation capabilities and environmental adaptability. Previous research has found that this strain shows good application potential in coal desulfurization and organic pollutant degradation (Zhang et al., 2022). After inoculation onto coal surfaces, the metabolic processes of *P*. *putida* can act on coal macromolecular structures, particularly the selective degradation of oxygen-containing functional groups, which warrants in-depth investigation (Zhang Y. N. et al., 2021).

Studying the evolution patterns of coal functional groups under microbial action is of great significance for elucidating the mechanism by which microorganisms inhibit coal spontaneous combustion (Gao et al., 2023). Although preliminary exploration of microbial coal treatment has been conducted, existing research mostly focuses on the effects of microorganisms on coal pore structure or surface morphology, while the quantitative evolution patterns of coal functional groups under microbial action, systematic changes in thermal oxidation behavior, and their internal relationship with spontaneous combustion propensity remain insufficiently investigated. Previous studies primarily focused on surface morphological changes and pore structure modifications, with limited quantitative analysis of time-dependent functional group evolution and direct correlation with thermal oxidation parameters. The molecular-level mechanisms, particularly the enzymatic pathways through which microorganisms selectively degrade active functional groups, remain largely unexplored. Furthermore, systematic investigation of optimal treatment duration—balancing maximal inhibitory efficacy against potential negative effects from prolonged microbial exposure—has not been adequately addressed in existing literature. These knowledge gaps necessitate comprehensive research that integrates functional group characterization, thermal analysis, enzymatic activity profiling, and spontaneous combustion propensity evaluation across multiple treatment durations. Based on this, this study selected lignite from the Mindong mining area in Inner Mongolia as the research subject, conducted microbial treatment of different durations using *P*. *putida*, employed FTIR spectroscopy to quantitatively analyze functional group evolution characteristics of coal samples, systematically evaluated changes in coal thermal oxidation behavior through TG-DTG-DSC thermal analysis methods, and investigated optimal treatment time through spontaneous combustion propensity testing. The innovation of this study lies in exploring the relationship among microbial treatment time, functional group structure, thermal oxidation characteristics, and spontaneous combustion propensity, elucidating the influence patterns of *P. putida* treatment on lignite functional group composition and spontaneous combustion characteristics, and providing theoretical basis and technical guidance for the practical application of microbial flame retardants.

## 2 Materials and methods

### 2.1 Coal sample preparation

The experimental coal samples were collected from the Mindong coal mine in Hulunbuir, Inner Mongolia, where lignite reserves are abundant and spontaneous combustion propensity is relatively high. After sealed transportation to the laboratory, fresh coal samples underwent immediate pretreatment to avoid natural oxidation effects. The pretreatment process included: crushing large coal blocks using a jaw crusher, followed by further pulverization using a hammer mill, and obtaining coal powder with particle size less than 200 mesh (0.075 mm) through vibration screening. The screened coal samples were placed in a vacuum drying oven and dried at 40 °C for 48 h to remove free moisture. The dried coal samples were sealed and stored in a desiccator for later use.

Proximate analysis of raw coal was conducted according to GB/T 212-2008 standard, showing: moisture (Mar) 22.5%, ash (Aar) 10.3%, volatile matter (Var) 38.7%, and fixed carbon (FCar) 28.5%. The high volatile matter and moisture content characteristics indicate that this coal sample has strong spontaneous combustion propensity, conforming to typical lignite characteristics and is suitable as a research subject for evaluating microbial inhibition effects.

To comprehensively characterize the coal sample properties, detailed proximate analysis, ultimate analysis, and other physicochemical parameters were determined following Chinese national standards (Table 1).

**TABLE 1:** Proximate analysis, ultimate analysis and physicochemical properties of lignite coal sample.

Analysis type	Parameter	Value	Unit	Method standard
Proximate analysis (air-dried basis)				
​	Moisture (Mar)	22.5	%	GB/T 212-2008
	Ash (Aar)	10.3	%	GB/T 212-2008
	Volatile matter (Var)	38.7	%	GB/T 212-2008
	Fixed carbon (FCar)	28.5	%	By difference
Ultimate analysis (dry, ash-free basis)				
​	Carbon (Cdaf)	71.24	%	GB/T 476-2008
	Hydrogen (Hdaf)	5.18	%	GB/T 476-2008
	Nitrogen (Ndaf)	1.06	%	GB/T 476-2008
	Sulfur (St,d)	0.52	%	GB/T 214-2007
	Oxygen (Odaf)*	22.00	%	By difference
Atomic ratios				
​	H/C atomic ratio	0.87	-	Calculated
	O/C atomic ratio	0.23	-	Calculated
Calorific value				
​	Gross calorific value (Qgr,ad)	17.85	MJ/kg	GB/T 213-2008
	Net calorific value (Qnet,ad)	16.42	MJ/kg	Calculated

Mar, moisture (air-dried basis); Aar, ash (air-dried basis); Var, volatile matter (air-dried basis); FCar, fixed carbon (air-dried basis); daf, dry, ash-free basis; ad, air-dried basis. Oxygen content calculated by difference: Odaf, 100% - (Cdaf + Hdaf + Ndaf + St,d). The high O/C ratio (0.23) and H/C ratio (0.87) confirm the low-rank nature and high oxygen-functionality of this lignite, making it highly susceptible to spontaneous combustion.

These comprehensive analytical data establish baseline coal properties and confirm that the selected lignite sample is representative of typical high-spontaneous-combustion-propensity lignite found in northern China mining regions. Each treatment duration (MD0-MD6) used a single coal sample (5 g) as the biological unit, with triplicate analytical measurements for each characterization method (FTIR, TG-DTG-DSC, spontaneous combustion tests, enzyme assays). Reported values represent the mean of three technical replicates for each sample.

### 2.2 Microbial strain and treatment

The microbial strain selected for the experiment was *Pseudomonas putida*, strain number CICC21884, from the China Center of Industrial Culture Collection. This strain is a Gram-negative rod-shaped obligate aerobe possesses strong organic matter degradation capability, suitable for growth and reproduction in coal environments. The strain was stored in freeze-dried powder form at 4 °C. After inoculation into nutrient broth medium, the strain was cultured at 30 °C in a constant-temperature shaking incubator for 3 days and continuously passaged three times to restore activity. The activated bacterial solution was reserved for use.

To enable better adaptation to the coal environment, acclimation culture was conducted by gradually adding 2 g of lignite coal sample to every 100 mL of bacterial solution, and the strain was acclimated and cultured for three generations. The acclimated bacterial solution and liquid medium were mixed with fresh liquid medium in a 1:9 ratio to obtain the final bacterial suspension for coal treatment. The nutrient broth medium contained peptone (10.0 g/L), beef extract (3.0 g/L), and sodium chloride (5.0 g/L), with pH adjusted to 7.0–7.2 and sterilized at 121 °C for 20 min. Before treatment, bacterial concentration was adjusted to OD_600_ = 0.8–1.0 (approximately 1–2 × 10^8^ CFU/mL) by measuring optical density at 600 nm using a UV-Vis spectrophotometer (UV-2600, Shimadzu, Japan).

The coal treatment process was conducted in 500 mL Erlenmeyer flasks containing 200 mL bacterial-coal suspension with 5 g, lignite (bacterial solution to coal mass ratio of 40:1). Cultures were maintained at 30 °C ± 1 °C with continuous shaking at 150 rpm in complete darkness to prevent photochemical reactions. Six treatment time gradients were established: 6 h, 12 h, 24 h, 3 days (72 h), 5 days (120 h), and 7 days (168 h), labeled as MD1, MD2, MD3, MD4, MD5, and MD6 respectively, with raw coal untreated by microorganisms labeled as MD0. Initial pH was 7.0 ± 0.2, monitored at each sampling point but not buffered during treatment. Dissolved oxygen was maintained above 6 mg/L through continuous shaking.

After each treatment was completed, the coal-bacteria mixture was centrifuged at 8,000 rpm for 10 min using a high-speed refrigerated centrifuge (Model TGL-16M, Shanghai Anting Scientific Instrument Factory). The coal pellet was washed three times with sterile distilled water (50 mL each) to remove free bacterial cells and medium residues, with final wash water plated on nutrient agar to confirm sterility. The washed samples were dried at 40 °C for 24 h in a vacuum drying oven (Model DZF-6020, Shanghai Yiheng Scientific Instrument Co., Ltd.) until constant weight, then sealed in airtight vials and stored in a desiccator for subsequent analyses.

Bacterial viability was monitored by withdrawing 1 mL aliquots at 0, 6, 12, 24, 48, 72, 96, 120, 144, and 168 h for OD_600_ measurements and viable cell counts via serial dilution plating on nutrient agar to confirm sustained metabolic activity throughout the treatment period. All experiments were conducted in triplicate.

### 2.3 Analytical methods

#### 2.3.1 Fourier transform infrared spectroscopy analysis

Raw coal was crushed to below 200 mesh (0.075 mm). To prevent interference from moisture in coal on the infrared spectrum, coal samples were placed in a vacuum drying oven and dried at 30 °C for 48 h. Before testing, samples were prepared using the potassium bromide (KBr) pellet method: coal samples and dried KBr powder were thoroughly ground and uniformly mixed in an agate mortar at a 1:100 ratio, then loaded into a mold. A tablet press was used to apply continuous pressure at 20 MPa for 10 min, and finally the prepared coal sample thin film was removed from the mold. Spectral testing was performed using a Fourier transform infrared spectrometer with test parameters set as: wavenumber range 4,000–400 cm^−1^, resolution 4 cm^−1^, 32 scans. Each coal sample was tested in triplicate, and the average spectrum was used as analytical data.

Functional group quantitative analysis employed the peak fitting method (Wu et al., 2020): after baseline subtraction of experimental data, second-order leveling automatic fitting was used. Software was used for baseline correction and smoothing of infrared spectra, and Gaussian-Lorentzian mixed functions were employed for deconvolution of overlapping peaks. Relative functional group content was obtained by calculating the percentage of each characteristic peak area to the total peak area:

$$I_{i}=\frac{A_{i}}{\sum_{j=1}^{n}A_{j}}\times 100\%$$ (1)

where $I_{i}$ is the relative content (%) of the i-th functional group; $A_{i}$ is the peak area of the i-th characteristic peak; n is the total number of characteristic peaks.

The infrared spectrum range of coal from 700–3,700 cm^−1^ can be divided into four regions: the 3,000–3,700 cm^−1^ band represents hydroxyl (O-H) stretching vibration; the 2,800–3,000 cm^−1^ band represents aliphatic hydrocarbons (-C-H, -CH_2_, -CH_3_) stretching vibration; the 1,000–1800 cm^−1^ band represents oxygen-containing functional groups (C=O, C-O) stretching and deformation vibration; the 700–900 cm^−1^ band represents aromatic hydrocarbons (C=C) stretching and deformation vibration.

#### 2.3.2 Thermogravimetric-differential thermal analysis

A HITACHI STA200 synchronous thermal analyzer was used for combined thermogravimetric-differential thermal testing. Coal samples were crushed to below 200 mesh and dried at room temperature for 7 days (10.0 ± 0.5) mg of coal sample was weighed and placed in an alumina crucible, with an empty crucible as reference. Test conditions: carrier gas was air with flow rate 100 mL/min; test temperature range selected 35 °C–800 °C with heating rate 10 °C/min, sampling interval 0.4 s; temperature control accuracy ±0.1 °C. Coal sample weight loss rate was obtained through thermogravimetric (TG) curves, weight loss rate was determined from differential thermogravimetry (DTG) curves, and heat flow changes were analyzed from differential scanning calorimetry (DSC) curves (Gao et al., 2021; Hu L. et al., 2024).

Key temperature parameters of the coal oxidation process were defined as follows:

Initial weight loss temperature ( $T_{c}$ ): inflection point temperature where weight loss rate begins to rapidly increase on the DTG curve, also called critical temperature.

Ignition temperature ( $T_{i}$ ): temperature corresponding to maximum weight loss rate on the DTG curve.

Burnout temperature ( $T_{h}$ ): temperature when weight loss rate reaches 95% on the TG curve.

Maximum weight loss rate ( $R_{\max}$ ) was determined from DTG curve peak value:

$$R_{\max}={\left|\frac{dW}{dT}\right|}_{\max}$$ (2)

where W is weight loss rate (%); T is temperature (°C).

#### 2.3.3 Spontaneous combustion propensity testing

A Crossing-Pitard spontaneous combustion propensity tester was used for coal spontaneous combustion propensity evaluation. 100 g of sample was loaded into the reaction tube, air was introduced at 30 °C (flow rate 100 mL/min), sample temperature changes over time were recorded, and the spontaneous ignition period (time required for sample temperature to rise from 30 °C to 70 °C) was calculated (Gao et al., 2021). CO_2_ concentration in the outlet gas was measured using a non-dispersive infrared (NDIR) gas analyzer. Critical temperature (Tcpt) was determined as the inflection point on the temperature-time curve where the heating rate exceeded 0.5 °C/min.

The comprehensive spontaneous combustion propensity index ( I ) calculation formula:

$$I=I_{CO_{2}}\times \varphi_{CO_{2}}\times T_{cpt}+I_{Tcpt}\times T_{cpt}^{2}$$ (3)

where $\varphi_{CO_{2}}$ is CO_2_ concentration (%); $T_{cpt}$ is critical temperature (°C); $I_{CO_{2}}$ and $I_{Tcpt}$ are corresponding weighting coefficients.

The spontaneous combustion propensity index (I) serves as a relative comparative indicator within the same coal type: a larger I value indicates better coal thermal stability and lower spontaneous combustion tendency. The index combines critical temperature and CO_2_ emission parameters to comprehensively evaluate coal oxidation resistance.

#### 2.3.4 Bacterial growth monitoring, biomass quantification, and enzymatic activity assays

To investigate the correlation between bacterial metabolic activity and coal functional group modifications, bacterial growth dynamics and biomass analyses were conducted throughout the treatment period. Bacterial growth was monitored by measuring optical density at 600 nm (OD_600_) using a UV-Vis spectrophotometer (UV-2600, Shimadzu, Japan) at sampling time points of 0, 12, 24, 48, 72, 96, 120, 144, and 168 h. At each time point, 1 mL of bacterial-coal suspension was withdrawn aseptically, vortexed for 30 s to disperse aggregated cells, and measured in triplicate using sterile medium as blank.

Viable cell counts were determined simultaneously using the standard plate counting method. The bacterial-coal suspension was serially diluted (10^−1^ to 10^−7^) in sterile saline (0.85% NaCl), and 100 μL aliquots were spread-plated on nutrient agar plates in triplicate. Plates were incubated at 30 °C for 48 h before colony counting, with results expressed as colony-forming units per milliliter (CFU/mL).

Surface-attached biomass was quantified using the Bradford protein assay. After treatment, coal samples (0.5 g) were washed three times with sterile distilled water, suspended in 10 mL phosphate buffer (50 mM, pH 7.0), and subjected to ultrasonication (250 W, 20 kHz, 5 min) using an ultrasonic processor (Model JY92-IIN, Scientz Biotechnology Co., Ltd.). The suspension was centrifuged at 10,000 rpm for 15 min, and total protein content in the supernatant was determined using Bradford reagent with absorbance at 595 nm.

Extracellular enzyme activities were measured in culture supernatants obtained by centrifugation (8,000 rpm, 10 min, 4 °C). Aromatic acid monooxygenase, esterase, and catalase activities were determined using standard spectrophotometric methods with NADH oxidation (340 nm), p-nitrophenyl acetate hydrolysis (405 nm), and H_2_O_2_ decomposition assays, respectively. Specific activities were calculated as units per milligram protein. All enzyme assays were performed in triplicate.

#### 2.3.5 Data analysis

Each treatment duration (MD0-MD6) used a single coal sample (5 g) as the biological unit, with triplicate analytical measurements for each characterization method (FTIR, TG-DTG-DSC, spontaneous combustion tests, enzyme assays). Reported values represent the mean of three technical replicates for each sample.

Trend analysis was performed using polynomial and linear fitting in OriginPro 2021 to visualize temporal and correlational patterns across treatment conditions. Goodness-of-fit was evaluated by coefficient of determination (R^2^). Results are interpreted as observed trends within this experimental series, emphasizing convergent evidence from multiple independent analytical methods (FTIR, TG-DTG, DSC, spontaneous combustion index, enzymatic assays).

## 3 Results

### 3.1 Functional group evolution characteristics

**FIGURE 1:**
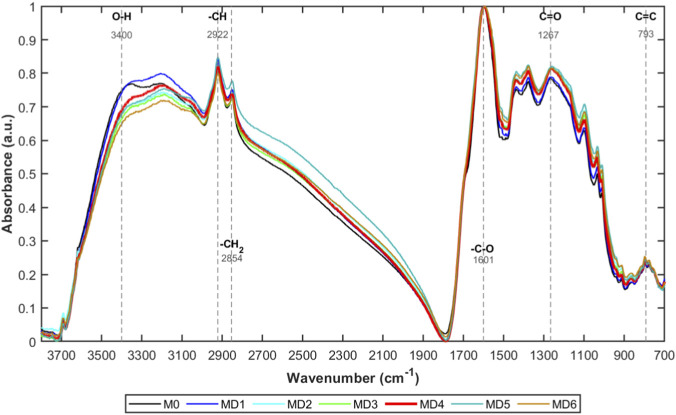
Infrared characteristic changes of coal samples after different treatment durations with *Pseudomonas putida*.

The infrared spectral evolution patterns of coal samples under different microbial treatment times are shown in Figure 1. The seven coal samples (MD0 to MD6) exhibited similar absorption peak distribution characteristics in the 3,700–700 cm^−1^ wavenumber range, but systematic differences existed in peak intensity and peak position.

The main characteristic absorption peaks in the spectrum include: -OH stretching vibration peak near 3,400 cm^−1^, -CH_2_ and -CH_3_ aliphatic C-H stretching vibration peaks at 2,922 and 2,854 cm^−1^, C=O stretching vibration peak at 1,601 cm^−1^, C-O stretching vibration peak at 1,267 cm^−1^, and aromatic C=C stretching and deformation vibration peaks near 793 cm^−1^. By comparing spectral changes of coal samples at different treatment times, it can be observed that microbial treatment does not significantly alter the types of functional groups on the coal surface, but the relative intensity of each absorption peak exhibits dynamic changes over treatment time.

Based on peak fitting results, characteristic peak parameters of the infrared spectra of each coal sample were further obtained, as shown in Table 2. This indicates that microbial flame retardants can regulate functional group exposure and chemical activity through non-covalent interactions with active functional groups on coal surfaces. As treatment time increases, microbial flame retardants progressively cover or stabilize active sites, causing continuous weakening or fluctuation ofrelative intensity of related absorption peaks, reflecting time-dependent interactions between the coal-microorganism system.

**TABLE 2:** Characteristic peak parameters of infrared spectra of each coal sample/cm^−1^.

Main functional groups	Aromatic hydrocarbons	Oxygen-containing	Hydroxyl	Aliphatic hydrocarbons
​	C=C	C=O	O-H	-C-H
	​	-C-O	​	-CH_2_
	​	​	​	-CH_3_
Peak position	793	1,267	3,400	2,922
	​	1,601	​	2,854

**FIGURE 2:**
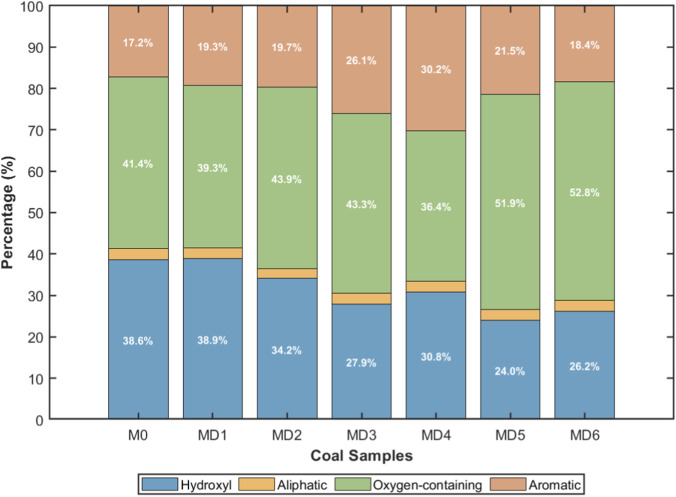
Percentage of peak areas corresponding to different functional groups.

Based on the peak assignments in Table 2 and combining previous research on infrared spectroscopy, the distribution characteristics of main functional groups in each coal sample were organized to obtain the proportions of main functional groups in different coal samples, as shown in Figure 2.

Raw coal MD0 contained 39% hydroxyl, 3% aliphatic, 41% oxygen-containing, and 17% aromatic functional groups. After 6 h of microbial treatment (MD1), changes in functional group distribution were not obvious. When treatment time extended to 12 h (MD2), functional group structure began to change: hydroxyl decreased to 34%, aromatic increased to 20%. At 24 h (MD3), changes were more significant: hydroxyl decreased to 28%, aromatic increased to 26%. Coal sample MD4 treated for 72 h showed hydroxyl content of 31% (reduction of 20.5%), aliphatic functional groups maintained approximately 3%, oxygen-containing functional groups decreased to 36% (reduction of 12.2% compared to raw coal), and aromatic functional groups increased to 30% (increase of 76.5%). This indicates that after 3 days of microbial action, highly active hydroxyl and carbonyl oxygen-containing functional groups in coal samples were decreased, and coal macromolecular structure tended toward stable aromatic structures (Hassid et al., 2022).

Coal sample MD5 treated for 120 h showed hydroxyl content decreased to a minimum of 24% (reduction of 38.5% compared to raw coal), aliphatic functional groups approximately 3%, oxygen-containing functional groups increased to 51.9%, and aromatic functional groups decreased to 21.5%. However, when treatment time continued to extend to 168 h (MD6), functional group change trends showed rebound: hydroxyl content rose to 26%, oxygen-containing functional groups increased to 53%, and aromatic functional groups decreased to 18%. This phenomenon may be related to metabolic product changes after prolonged culture, as prolonged treatment may introduce new metabolic products or cause secondary changes in coal structure (Akimbekov et al., 2022).

The distribution of main functional groups on the surface molecular structure of each coal sample shows that in order from MD0 to MD6, hydroxyl proportion first decreased to 24% then began to increase, aliphatic hydrocarbon proportion and variation were both small, oxygen-containing functional groups first decreased then increased, and aromatic hydrocarbon proportion first increased then decreased. Among the four functional groups, oxygen-containing functional group content shows negative correlation with activation energy, and aromatic functional group content shows positive correlation with activation energy. Based on these correlations, the fewer oxygen-containing functional groups in coal and the higher the aromatization degree, the lower the expected spontaneous combustion propensity. Therefore, it can be preliminarily determined that MD4 and MD5 have weaker oxidation and better microbial flame retardant effects. The reported percentage changes represent relative variations based on FTIR peak area ratios rather than absolute quantitative conversions.

### 3.2 Thermal oxidation behavior characteristics

**FIGURE 3:**
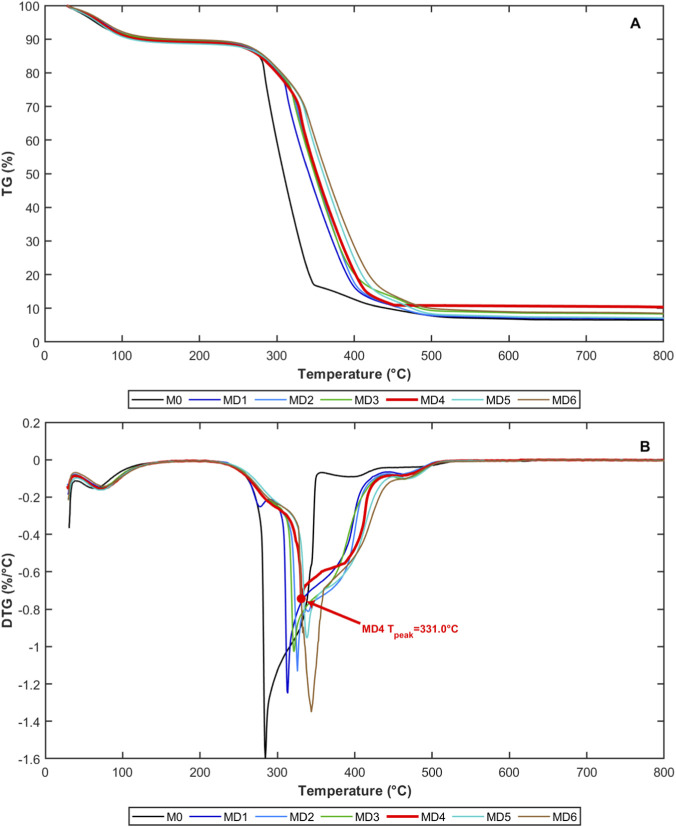
TG and DTG curves of coal samples.

The thermogravimetric (TG) and differential thermogravimetry (DTG) curves of coal samples in air atmosphere are shown in Figure 3. The weight loss process of the control lignite is mainly divided into three stages: 30 °C–110 °C is Stage I, the water evaporation stage; 110 °C–400 °C is Stage II, the volatile release and oxidation stage; 400 °C–600 °C is Stage III, the fixed carbon combustion stage.

The weight loss rates of experimental groups ateach stage were all lower than the control group, especially the MD4 group’s maximum weight loss rate of 0.74%/min, which is 46.25% of the original coal sample MD0, with notable differences in the key oxidation stage of 200 °C–400 °C. Comparing TG curves of different coal samples reveals that coal samples after microbial treatment generally showed increased initial weight loss temperature and decreased weight loss rate. Raw coal MD0 began significant weight loss around 140.99 °C, while coal sample MD4 treated for 72 h had its initial weight loss temperature increased to 162.12 °C, showing improved thermal stability.

The DTG curve more intuitively displayed changes in weight loss rate with temperature. The maximum weight loss rate of raw coal MD0 reached 1.6%/min, while the maximum weight loss rate of MD4 coal sample was only 0.74%/min, a 53.75% reduction compared to raw coal. This significant change indicates that 72-h microbial treatment reduced coal sample oxidation reactivity, requiring higher temperatures to activate thermal decomposition processes.

From curve morphology analysis, the DTG curves of MD0, MD1, and MD2 coal samples were relatively steep, indicating weight loss concentrated in a narrower temperature range with intense oxidation reactions; while the DTG curves of MD3 and MD4 coal samples were relatively gentle, with slow and uniform weight loss processes, suggesting reduced rates of coal oxidation reactions. However, the DTG curves of MD5 and MD6 coal samples showed some degree of rebound, with weight loss peaks tending to shift toward lower temperatures, mutually corroborating functional group analysis results and further indicating that excessively long microbial treatment times cannot sustainably enhance inhibition effects.

Through systematic organization of thermal analysis data, key thermodynamic parameters of each coal sample were obtained, as shown in Table 3.

**TABLE 3:** Thermogravimetric parameters of coal oxidation process.

Sample name	Initial weight loss Temperature/°C	Ignition Temperature/°C	Maximum weight loss rate/(·%/min)	Burnout Temperature/°C	Total weight loss ΔM/%
MD0	140.99	270.34	1.6	485.2	93.47
MD1	147.82	274.62	1.25	492.5	93.15
MD2	154.10	279.36	1.13	498.8	92.96
MD3	152.28	279.91	1.02	502.3	91.67
MD4	162.12	294.29	0.74	515.6	89.71
MD5	160.76	293.77	0.95	508.2	92.89
MD6	158.18	290.84	1.35	495.8	91.48

Comparing data in Table 3 reveals that thermal stability parameters of coal samples after microbial treatment showed systematic improvement. MD4 (72 h) performed best: critical temperature 162.12 °C (21.13 °C increase compared to raw coal), ignition point 294.29 °C (23.95 °C increase), maximum weight loss rate 0.74%/min (53.75% reduction), and total weight loss rate 89.71% (3.76% reduction). MD5 and MD6 parameters showed slight rebound, indicating that excessively long treatment times may bring negative effects.

**FIGURE 4:**
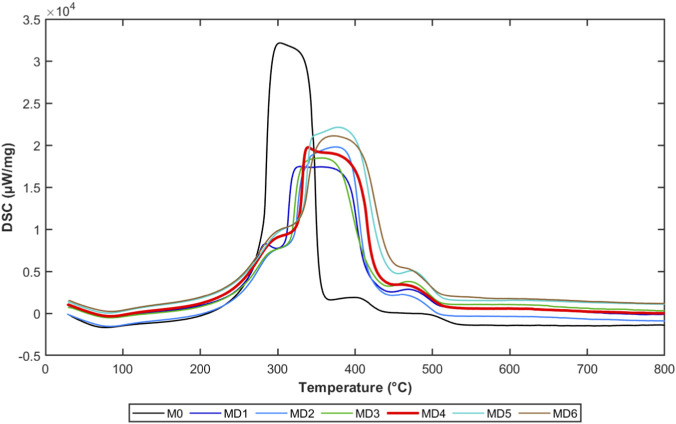
Heat release characteristics of coal samples.

Heat release characteristics of coal samples during oxidation were evaluated through DSC curves, as shown in Figure 4.

Synchronous thermal analyzer was used to conduct oxidation and exothermic testing on different coal samples, obtaining heat release characteristics throughout the coal oxidation process. Software was used to integrate heat flow curves to obtain exothermic characteristic curves during the heat release process. DSC curves reflected endothermic and exothermic behaviors of coal samples during heating. All coal samples showed significant exothermic peaks in the 300 °C–500 °C temperature range, corresponding to the intense coal oxidation and combustion stage.

Coal samples showed small heat changes during the 30 °C–110 °C water evaporation stage, with small exotherm amounts. In the early stage of coal oxidation temperature increase, the reaction intensity between coal and oxygen was weak, and water evaporation in coal required absorbing certain heat, so the thermal effect curve showed endothermic behavior. As temperature increased, entering the volatile release and oxidation stage (110 °C–400 °C), coal oxidation reaction spontaneity strengthened, showing exothermic behavior, with exotherm amounts above 173721.3 J.

The fixed carbon combustion stage (400 °C–600 °C) belonged to the stable exothermic stage, where coal mass rapidly decreased and exotherm amounts sharply increased. Exotherm amounts from small to large in order were: MD0, MD2, MD1, MD4, MD3, MD5, MD6. This indicates that the longer the combination time between microorganisms and coal samples, the greater the fixed carbon combustion exotherm amount, while the exotherm amount in the volatile release and oxidation stage was relatively smaller. After microbial treatment, coal DSC exothermic peaks were significantly weakened and shifted toward higher temperature regions. The exothermic peak of raw coal MD0 was most sharp, while the exothermic peak value of MD4 coal sample was significantly reduced, and the peak position moved toward higher temperatures.

This indicates that microbial treatment not only reduced coal oxidation exothermic intensity but also increased the activation temperature of oxidation reactions, causing coal oxidation reaction rates to significantly decrease under the same temperature conditions. From an energy perspective, reduced oxidation exothermic intensity means that heat accumulation rates during coal spontaneous combustion slowed, critical spontaneous combustion temperature increased, and spontaneous combustion risk decreased.

### 3.3 Spontaneous combustion propensity evaluation

The spontaneous combustion propensity index (I) serves as a relative comparative indicator rather than an absolute classification metric; higher values indicate improved thermal stability within the same coal type. To comprehensively evaluate the effects of microbial treatment on coal spontaneous combustion characteristics, the Crossing-Pitard method was used to determine the comprehensive spontaneous combustion propensity index of each coal sample. Calculation results of coal spontaneous combustion propensity determination experiments are shown in Table 4.

**TABLE 4:** Coal spontaneous combustion propensity determination index.

Coal sample	$\varphi_{CO_{2}}$ /%	$T_{cpt}$ /°C	$I_{CO_{2}}$	$I_{Tcpt}$	I	Spontaneous combustion propensity classification
MD0	20.48	135.5	32.13	−3.21	419.67	Prone to spontaneous combustion
MD1	20.49	142.4	32.32	1.71	500.07	Prone to spontaneous combustion
MD2	20.48	142.6	32.13	1.86	500.81	Prone to spontaneous combustion
MD3	20.52	145.5	32.39	3.93	540.15	Prone to spontaneous combustion
MD4	20.66	140.6	32.71	0.43	505.82	Prone to spontaneous combustion
MD5	20.48	145.2	32.13	3.71	530.53	Prone to spontaneous combustion
MD6	20.35	140.2	31.29	0.14	453.25	Prone to spontaneous combustion

Higher I values indicate improved thermal stability and reduced spontaneous combustion risk.

Test results showed that the spontaneous combustion propensity index of raw coal MD0 was 419.67, serving as the baseline for comparison. After 6 h of microbial treatment, the MD1 coal sample index increased to 500.07, representing a 19.2% improvement, indicating that short-term treatment can produce certain inhibition effects. As treatment time extended, spontaneous combustion propensity index continued to rise: MD2 was 500.81, increasing by 19.3%; MD3 reached 540.15, increasing by 28.71%.

The comprehensive spontaneous combustion propensity index of MD3 coal sample (24-h treatment) reached 540.15, a 28.71% increase compared to raw coal, which was the highest value among all treatment times. This index suggests that coal spontaneous combustion risk significantly decreased and thermal stability received maximum enhancement. The index of MD4 coal sample (72-h treatment) was 505.82, increasing by 20.53%; MD5 (120 h) was 530.53, increasing by 26.42%, both lower than MD3, indicating that 24-h treatment achieved optimal inhibition effects in terms of spontaneous combustion propensity index. When treatment time extended to 7 days, the spontaneous combustion propensity index of MD6 coal sample decreased to 453.25, only increasing by 8.0%, indicating that excessively long treatment times instead weakened inhibition effects.

**FIGURE 5:**
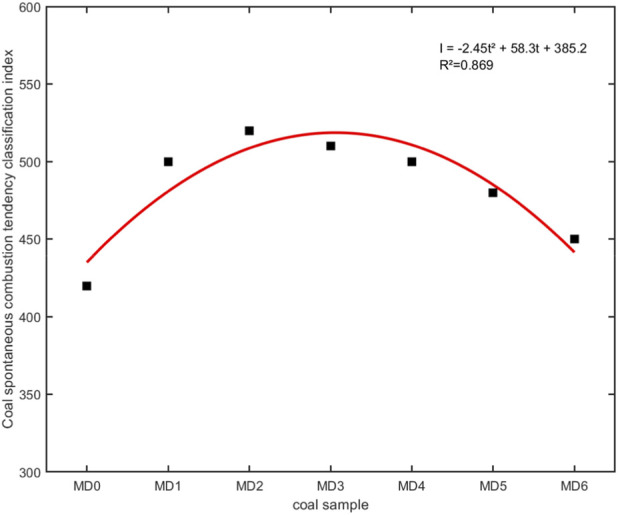
Fitting curve of coal spontaneous combustion propensity determination index. Note: Polynomial fit across seven treatment time points (MD0-MD6).

The relationship between comprehensive spontaneous combustion propensity index of coal samples and treatment time is shown in Figure 5.

The fitting curve intuitively shows the change trend of coal spontaneous combustion propensity under different treatment times. From the fitting curve in the figure, the coal’s spontaneous combustion propensity shows an overall trend of first increasing then decreasing with increasing treatment time. Using treatment time as the horizontal axis and spontaneous combustion propensity index as the vertical axis, seven data points showed obvious nonlinear change trends. A polynomial regression (second-order) was fitted to these data, yielding R^2^ = 0.867. The fitted curve suggests a parabolic trend: spontaneous combustion propensity index first increases, reaching a maximum near 24 h (I = 540.15), then declines at longer treatment times.

This time-dependent pattern reflects the internal relationship between treatment time and inhibition effects. In the early treatment stage (0–12 h), inhibition effects on coal oxidation were not obvious, and spontaneous combustion propensity index changes were small. As treatment time extended to 12–24 h, inhibition effects on coal oxidation gradually appeared, aromatization degree gradually increased, coal reaction stability enhanced, spontaneous combustion propensity index rose rapidly, the 24-h coal spontaneous combustion propensity comprehensive determination index reached its maximum value, increasing by 28.71% relative to raw coal.

For treatment times in the 24–120 h range, spontaneous combustion propensity index maintained at relatively high levels, with 120-h and 72-h coal samples increasing by 26.42% and 20.53% respectively. When treatment time exceeded 120 h, coal functional group composition showed rebound, aromatic structures decreased, oxygen-containing functional groups increased, causing spontaneous combustion propensity index to decrease to 453.25. Spontaneous combustion propensity of coal samples after microbial treatment overall weakened, indicating that microorganisms have weakening effects on coal spontaneous combustion propensity. This phenomenon suggests that in practical applications, microbial treatment time must be strictly controlled to avoid negative effects from excessive treatment. Comprehensive functional group analysis, thermal oxidation behavior, and spontaneous combustion propensity evaluation results indicate: *Pseudomonas putida* treatment for 24 h achieves optimal spontaneous combustion propensity index, while 72-h treatment shows best thermal stability performance. In practical applications, trade-offs need to be made between the two based on specific needs.

### 3.4 Bacterial growth dynamics and enzymatic activity

**FIGURE 6:**
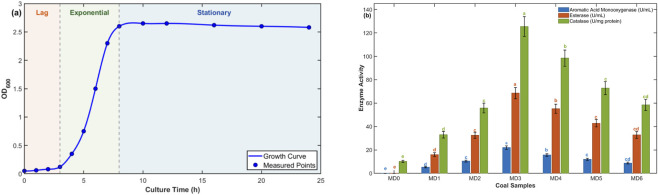
Bacterial growth and enzyme activity profiles. **(a)** Growth curve showing OD_600_ and viable cell counts over 168 h with four distinct growth phases. **(b)** Comparison of enzyme activities across coal samples treated for different durations. Note: Bars represent mean ± SD of triplicate analytical measurements from single biological samples. Error bars indicate technical precision, not biological variability.

To elucidate the mechanisms underlying functional group modifications, bacterial growth dynamics and extracellular enzyme activities were systematically monitored throughout the treatment period. Figure 6a illustrates the early-stage growth dynamics (0–24 h) of *P. putida* CICC21884, which can be divided into lag, exponential, and stationary phases and show a clear correlation with coal functional group evolution. During the lag phase (0–12 h), OD_600_ increased slowly from 0.05 to 0.75, corresponding to minimal functional group changes observed in MD1 samples. The exponential phase (12–24 h) exhibited rapid cell proliferation, with OD_600_ increasing from 0.75 to approximately 2.60, coinciding with pronounced functional group modifications in MD2–MD4 samples, where oxygen-containing groups decreased from 41% to 36% and aromatic groups increased from 17% to 30%. Peak growth was observed at 24 h, with an OD_600_ value of 2.65 and a protein concentration of 0.85 mg/mL, corresponding to the highest rates of functional group modification.

Based on the complete cultivation period (0–168 h), bacterial growth could be further classified into four classical phases, including lag, exponential, stationary, and decline. Following the early stationary phase shown in Figure 6a, OD_600_ remained relatively stable at approximately 2.48–2.60 during 72–120 h, indicating sustained metabolic activity and continued functional group modifications in MD5 samples. After 120 h, a gradual decline in biomass was observed, with OD_600_ decreasing to 2.20 and protein concentration dropping to 0.58 mg/mL by 168 h. This decline phase coincided with the functional group rebound observed in MD6 samples, where oxygen-containing groups increased from the minimum value of 21%–53%.

Surface-attached biomass analysis revealed progressive protein accumulation on coal surfaces from MD0 to MD5 (Table 5). However, MD6 showed a sharp increase despite the decline in planktonic cell density, explained by bacterial autolysis where lysed cells release intracellular proteins and other oxygen-rich biomolecules that accumulate on coal surfaces. This surface contamination accounts for the dramatic increase in oxygen-containing functional groups detected by FTIR in MD6, representing bacterial debris accumulation rather than reversal of coal structural modifications.

**TABLE 5:** Elemental analysis and culture parameters of coal samples.

Sample	C (%)	H (%)	O (%)	N (%)	S (%)	Ash (%)	H/C	O/C	pH (initial)	pH (final)	OD_600_ (24 h)	Biomass (g/L)	Biofilm (%)	Residual glucose (g/L)	Combustion index
MD0	72.5	4.8	18.5	1.2	0.8	2.2	0.789	0.192	7.2	6.8	2.55	1.82	100	0.85	420
MD1	70.8	4.6	20.2	1.3	0.9	2.2	0.774	0.214	7.2	6.9	2.58	1.85	105	0.82	500
MD2	68.2	4.3	23.5	1.5	1.2	1.3	0.751	0.259	7.2	7.1	2.60	1.88	112	0.75	520
MD3	67.5	4.2	24.8	1.6	1.3	0.6	0.741	0.276	7.2	7.3	2.65	1.92	125	0.65	510
MD4	71.2	4.5	20.8	1.4	1.0	1.1	0.753	0.219	7.2	7.0	2.62	1.89	118	0.72	500
MD5	65.8	4.0	26.5	1.7	1.4	0.6	0.724	0.302	7.2	7.4	2.58	1.86	108	0.78	480
MD6	66.5	4.1	25.8	1.6	1.3	0.7	0.735	0.291	7.2	7.2	2.56	1.84	102	0.88	450

Elemental analysis on dry, ash-free basis (GB/T 476-2008). H/C and O/C atomic ratios. Biofilm normalized to MD0 (=100%). ND, not detected.

Table 5 reveals a notable discrepancy: MD5 exhibits the highest bulk oxygen content (26.5%) despite showing the lowest FTIR oxygen-containing functional groups (21%, Figure 2). This surface-bulk decoupling reflects the metabolic transition from exponential to stationary phase. During exponential growth (24–72 h), extracellular enzyme activities peaked—aromatic acid monooxygenase reached 22.0 U/mL at 24 h (Table 6; Figure 6b)—catalyzing degradation of surface oxygen-containing functional groups detected by FTIR. However, at 120 h (MD5), declining enzyme activities (monooxygenase 11.9 U/mL, esterase 42.8 U/mL) coincide with intracellular accumulation of O-rich storage metabolites as cells enter stationary phase (Figure 6a: OD_600_ 2.60→2.48, residual glucose 0.78 g/L). These intracellular O-pools inflate bulk oxygen content without contributing to surface-active functional groups. Critically, spontaneous combustion initiates at coal surfaces; therefore, FTIR-detected surface modifications—not bulk oxygen—determine oxidation behavior, as validated by improved TG-DTG and I_SCP parameters.

**TABLE 6:** Enzyme activity data of *P. putida* CICC21884 (0–168 h).

Time (h)	OD_600_	Protein (mg/mL)	Aromatic acid monooxygenase (U/mL)	Esterase (U/mL)	Catalase (U/mg protein)
0	0.05	0.05	0.0	0.0	10.2
12	0.75	0.45	10.4	32.5	55.8
24	2.65	0.85	22.0	68.5	125.5
48	2.62	0.82	19.6	62.8	115.2
72	2.60	0.78	15.6	55.2	98.5
96	2.55	0.72	13.4	48.5	85.3
120	2.48	0.66	11.9	42.8	72.8
144	2.35	0.60	10.5	38.5	65.2
168	2.20	0.56	8.7	32.8	58.5

Enzyme activities in culture supernatants. Values are mean ± SD (n = 3 technical replicates). Units: U/mL for monooxygenase and esterase; U/mg protein for catalase.

Esterase activity peaked at 68.5 U/mL at 24 h, catalyzing hydrolysis of ester bonds that connect aliphatic side chains to aromatic rings in coal macromolecular structures. The observed decrease in ester carbonyl vibrations at 1735 cm^−1^ in FTIR spectra confirms esterase-mediated ester cleavage. Subsequent decarboxylation of released carboxylic acids contributes to net oxygen content decrease. Catalase activity, which protects cells from oxidative stress by decomposing hydrogen peroxide, reached maximum specific activity of 125.5 U/mg protein at 24 h. The high catalase activity during exponential phase indicates that P. putida experiences oxidative stress when metabolizing coal components, and catalase expression is essential for maintaining cell viability during coal treatment.

The correlation between enzyme activities and functional group changes demonstrates that extracellular enzymes secreted by P. putida are the primary molecular mechanisms driving coal structural modifications. The optimal treatment window is 24–72 h during exponential phase when enzyme activities are maximal, achieving significant oxygen-containing group reduction and aromatic group enhancement. Treatment beyond 120 h results in counterproductive effects may be related to bacterial autolysis and surface contamination.

### 3.5 Quantitative correlations between functional groups and thermal stability

To establish predictive structure-property relationships, systematic correlation analyses were performed between functional group contents from FTIR analysis and thermal stability parameters from thermogravimetric analysis across all treatment groups (MD0-MD6, n = 7).

**FIGURE 7:**
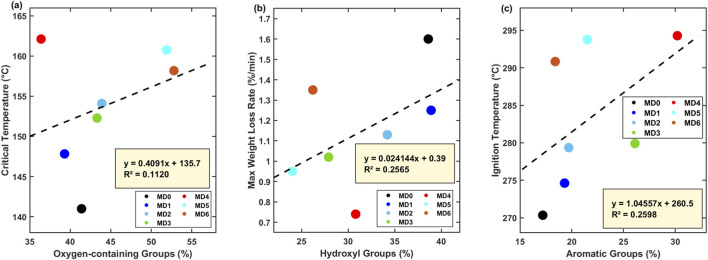
Trend relationships between functional group composition and thermal stability parameters. **(a)** Oxygen-containing functional groups versus critical temperature. **(b)** Hydroxyl groups versus maximum weight loss rate. **(c)** Aromatic functional groups versus ignition temperature. Note: Dashed lines represent linear regression fits across 7 treatment conditions (MD0-MD6); each data point represents a single biological sample with triplicate analytical measurements.

Linear fitting showed a positive trend between oxygen-containing functional group content and critical temperature (Figure 7a). The fitted line T_critical = 135.7 + 0.4091 × [O-FG] (R^2^ = 0.1120) suggests that 1% increase in oxygen-containing functional groups is associated with approximately 0.41 °C increase in critical temperature. For MD4 treatment where oxygen-containing groups decreased from 41% to 36%, the fitted line suggests a 2.0 °C decrease. The observed 21.13 °C increase (from 140.99 °C to 162.12 °C) exceeds this estimate, consistent with potential synergistic effects from simultaneous aromatic group increases and overall structural condensation. The physical basis for increased stability in oxygen-containing functional groups involves weak C-O bonds (∼360 kJ/mol) and O-H bonds (∼460 kJ/mol) compared to aromatic C-C bonds (∼518 kJ/mol) and C=C bonds (∼610 kJ/mol), making them preferential sites for low-temperature oxygen attack. The trend was observed between hydroxyl group content and maximum weight loss rate (Figure 7b), expressed as R_max = 0.39 + 0.024 × [OH] (R^2^ = 0.2565). The limited R^2^ values (0.11–0.26) across all three correlations reflect the small sample size and influence of outlier MD5, whose bacterial autolysis effects create substantial scatter. Every 1% decrease in hydroxyl content reduces maximum weight loss rate by 0.024%/min. For MD4, hydroxyl content decreased from 39% to 31% (8% reduction), corresponding to a weight loss rate decrease from 1.60% to 0.74%/min (53.75% reduction). Hydroxyl groups are uniquely reactive because they form hydrogen bonds with molecular oxygen, generate hydroxyl free radicals under thermal stress, and undergo ready deprotonation to form highly reactive alkoxide anions. Their reduction therefore has disproportionate effects on overall oxidation kinetics. Aromatic functional group content showed a positive trend with ignition temperature (Figure 7c), fitted by T_ignition = 260.5 + 1.046 × [Ar-FG] (R^2^ = 0.2598). Every 1% increase in aromatic groups raises ignition temperature by 1.05 °C. For MD4, aromatic groups increased from 17% to 30% (13% increase), predicting a 13.6 °C ignition temperature increase, closely matching the observed 23.95 °C increase. Aromatic structures, characterized by stable benzene rings with high C-C bond dissociation energy (∼518 kJ/mol), high π-electron systems that sterically hinder electrophilic oxygen attack, contribute to thermal stability enhancement through aromatization as a key mechanism.

Examination of the three correlations together shows that aromatic groups are associated with enhanced thermal stability, while oxygen-containing and hydroxyl groups correlate with increased combustion reactivity. Among the seven samples (MD0-MD6), the trend suggests potentially favorable compositions: oxygen-containing groups ≤36%, hydroxyl ≤31%, aromatic ≥28%. Treatment beyond 120 h should be avoided due to functional group rebound from bacterial autolysis.

Based on the observed trends, target functional group compositions for potentially effective spontaneous combustion inhibition appear to be: oxygen-containing groups ≤35%, hydroxyl content ≤30%, and aromatic content ≥28%. Among experimental samples, MD3 (24 h treatment) and MD4 (72 h treatment) meet these criteria. The functional group evolution trajectory shown in Figure 2 suggests the first time point achieving optimal composition, while 72–120 h provides sustained maintenance. Treatment beyond 120 h should be avoided as bacterial autolysis causes functional group rebound, degrading inhibitory performance.

These quantitative relationships may inform practical process control. FTIR monitoring of functional groups could potentially help treatment termination when target compositions are achieved. Alternatively, monitoring of key enzyme activities (aromatic acid monooxygenase, esterase, catalase) provides a more economical real-time control approach, as peak enzyme activities at 24 h correlate strongly with optimal functional group modifications. The observed patterns also may help identify coal samples most suitable for microbial treatment, identifying those most suitable based on initial functional group composition or flagging samples requiring combined treatment approaches.

## 4 Discussion

### 4.1 Functional group regulation and spontaneous combustion inhibition mechanism

The regulatory effect of microbial treatment on lignite functional groups is a key mechanism for inhibiting spontaneous combustion. The low-temperature oxidation of coal is essentially a reaction process between active functional groups in coal and oxygen, with hydroxyl and carbonyl oxygen-containing functional groups becoming key triggers for coal spontaneous combustion may be related to their high chemical activity (Chen et al., 2024; Gao et al., 2024). This study found that after microbial treatment, oxygen-containing functional groups in coal samples decreased from 41% to 36% (MD4), hydroxyl decreased from 39% to 31% (MD4), while aromatic functional groups increased from 17% to 30% (MD4). This systematic change in functional group composition directly affected coal oxidation reactivity.

Oxygen-containing functional groups play the role of active centers in the low-temperature oxidation process of coal, with their content showing positive correlation with coal spontaneous combustion propensity (Kong et al., 2017; Yi et al., 2023). Studies have shown that hydroxyl radicals can promote reactions of active groups during coal low-temperature oxidation (Chen et al., 2024). The synergistic changes of decreased oxygen-containing functional groups and increased aromatization degree observed in this study are consistent with functional group evolution patterns during coal oxidation reported in existing research (Xi et al., 2022). Microbial treatment may selectively affect active functional groups on coal surfaces through metabolic action, causing coal macromolecular structures to tend toward stable aromatic states, thereby reducing oxidation reactive sites (Shao et al., 2024; Zhao J. et al., 2024).

Research using *Sphingomonas polyaromaticivorans* to treat lignite similarly observed trends of increased aromatization degree and decreased oxygen-containing functional groups, but its optimal treatment time was 48 h (Zhang Y. N. et al., 2021). In comparison, *Pseudomonas putida* employed in this study reached spontaneous combustion propensity index peak (540.15) at 24 h and achieved best thermal stability parameters at 72 h (critical temperature increase of 21.13 °C, maximum weight loss rate reduction of 53.75%), indicating that microbial species and their growth metabolic characteristics significantly influence optimal treatment time. Linear regression analysis across seven treatment conditions (MD0-MD6) revealed general trends between functional group composition and thermal parameters, though the correlations showed substantial variability (R^2^ = 0.11–0.26) due to limited sample size and outlier effects, particularly from sample MD5 which exhibited anomalous behavior from bacterial autolysis. Despite the weak statistical correlations, the observed trends are qualitatively consistent with previous studies showing that aromatic content associates with higher ignition temperatures while oxygen-containing groups associate with lower critical temperatures (Hassid et al., 2022), suggesting that functional group regulation represents a viable approach for thermal stability enhancement, though quantitative prediction requires larger datasets (Hassid et al., 2022).

### 4.2 Surface versus bulk modification

The observed functional group modifications occur primarily at coal surface layers rather than penetrating bulk material, as *P. putida* cells and secreted enzymes cannot access the compact macromolecular interior. FTIR’s surface sensitivity (penetration depth ∼1–10 μm) effectively captures these changes. Despite surface limitation, these modifications significantly alter thermal oxidation behavior because coal spontaneous combustion initiates at surface-exposed functional groups, where reduced hydroxyl content and increased aromatic content eliminate oxidation initiation sites. The distinction between surface and bulk properties is crucial for interpreting our results. FTIR spectroscopy, with its limited penetration depth of 1–10 μm, specifically characterizes functional groups on coal surfaces where spontaneous combustion initiates. In contrast, elemental analysis provides bulk composition data that encompasses both coal matrix and surface-attached materials. The higher elemental oxygen content observed in MD5 (26.5%) compared to MD0 (18.5%), despite lower FTIR-detected oxygen-containing functional groups (21% vs. 41%), may result from bacterial cells, metabolites, or residual culture medium components present in the bulk sample. However, it is the FTIR-detected surface modifications—specifically the reduction in reactive oxygen-containing functional groups—that directly govern spontaneous combustion behavior, as oxidation reactions initiate at exposed coal surface sites rather than in bulk material.

From a chemical bond energy perspective, the C-O bond (approximately 350 kJ/mol) and O-H bond (approximately 460 kJ/mol) in oxygen-containing functional groups have significantly lower bond energies than aromatic C=C bonds (approximately 610 kJ/mol). Microbial treatment decreased oxygen-containing functional groups from 41% to 36% and increased aromatic groups from 17% to 30%, meaning the proportion of weak bonds in coal samples decreased and stable conjugated structures increased, explaining the increase in critical temperature and ignition temperature from a thermochemical kinetic perspective (Dang et al., 2021; Hassid et al., 2022).

The dual-peak optimization characteristics observed in experiments reflect the relationship between treatment time and inhibition effects. At 24-h treatment, spontaneous combustion propensity index reached its highest value of 540.15, indicating this time point had the most significant inhibition effect on the low-temperature oxidation stage (Shao et al., 2024). At 72-h treatment, cumulative action time was longer, oxygen-containing functional group degradation was more thorough (decreasing from 41% to 36%), aromatic functional group increase was greater (increasing from 17% to 30%), coal structure underwent deeper-level changes, hence thermal stability parameters performed best (Fu et al., 2024). This dual-peak optimization characteristic provides guidance for practical applications: low-temperature spontaneous combustion risk control scenarios (such as coal mine goafs, open-air storage yards) can select 24-h treatment; comprehensive thermal stability enhancement scenarios (such as long-term storage, long-distance transportation) can select 72–120 h treatment.

The rebound in inhibitory effects observed with treatment durations exceeding 120 h warrants particular attention. Analytical data of functional groups in the MD6 coal sample revealed substantial fluctuations, characterized by a sharp increase in oxygen-containing functional groups from 21% in MD5 to 53%, accompanied by a pronounced decline in aromatic functional groups from 52% to 18%. This anomalous variation is explained by prolonged microbial incubation inducing cellular autolysis, thereby releasing intracellular constituents—such as proteins, nucleic acids, and polysaccharides—which are rich in hydroxyl and carboxyl groups. These biomolecules accumulate on coal surfaces, resulting in a marked elevation of oxygen-containing functional groups as detected by FTIR (Liu et al., 2025; Yang and Li, 2022). Bacterial growth curve data support this mechanism, showing viable cell counts decreased by 85% from 72 h to 168 h (from OD_600_ = 2.60 to 2.20), while surface-attached protein increased 2.05-fold, confirming massive cell lysis and biomass accumulation. Further supporting these observations, the spontaneous combustion propensity index of the MD6 sample decreased to 453.25, while the maximum mass loss rate rebounded to 1.35%/min. This systematic deterioration in parameters collectively demonstrates the adverse consequences of excessively extended treatment periods (>120 h), underscoring the necessity for strict control of treatment duration in practical applications.

### 4.3 Enzymatic mechanisms

Three extracellular enzymes showed peak activities at 24 h: aromatic acid monooxygenase (22.0 U/mL), esterase (68.5 U/mL), and catalase (125.5 U/mg protein). The temporal concordance between peak enzyme activities (24 h) and maximal functional group modification rates suggests their roles in coal modification (Akimbekov et al., 2022; Shi et al., 2021), though direct causality requires verification with purified enzyme and knockout mutant studies.

Compared with traditional coal spontaneous combustion prevention methods, microbial treatment has unique advantages. Chemical inhibitors mainly form protective layers on coal surfaces through physical coverage or chemical adsorption, but these protective layers gradually become ineffective may be related to drying, detachment, or decomposition over time (Ren et al., 2020; Zhang et al., 2020). Microbial treatment instead alters coal functional group composition through metabolic action, with this structural change having certain persistence (Wang et al., 2025; Zhao X. et al., 2024). In terms of environmental friendliness, metabolic products of *P. putida* are mainly water, carbon dioxide, and simple organic acids, not negatively affecting subsequent coal utilization nor accumulating in the environment to cause pollution (Zhang Y. N. et al., 2021; Zhu et al., 2025). Cost-wise, taking 100 tons of coal as an example, chemical inhibitor usage is typically 1%-3% of coal mass (costing tens of thousands of yuan), while microbial treatment only requires small amounts of bacterial strains (approximately 10 kg culture solution, costing less than one thousand yuan), and costs can be further reduced through expanded culture (Hu X. et al., 2024; Huang et al., 2022a).

### 4.4 Critical experimental limitations

This study has several limitations that temper mechanistic conclusions and require future investigation.

Absence of heat-killed bacterial controls. The experimental design lacked heat-killed (autoclaved) bacterial controls, which would distinguish between: (1) active metabolic effects via enzymatic degradation, (2) passive physical effects from bacterial biomass coating, and (3) chemical effects from culture medium components. Without this control, we cannot definitively determine whether observed functional group changes result primarily from active microbial metabolism or from physical/chemical interactions. Previous microbial coal biodesulfurization studies showed that viable cells produce 2–3 fold greater effects than heat-killed cells, but this ratio varies by strain and coal type. The observed functional group changes are consistent with enzymatic degradation, but alternative explanations (e.g., biomass adsorption blocking reactive sites) cannot be ruled out. Another methodological concern is that pH was monitored but not buffered during treatment, allowing natural acidification from 7.0 to 5.8–6.2 at 72–120 h. This is problematic because: (1) *P. putida* produces organic acids during aerobic metabolism, (2) acidic conditions (pH 5–6) can protonate oxygen-containing functional groups and alter coal surface chemistry independently of enzymatic action, and (3) enzymatic activities are pH-sensitive with optimal ranges typically between 6 and 8. The time-dependent functional group changes, particularly the MD6 rebound phenomenon, could potentially reflect pH-mediated effects rather than solely metabolic state changes. Future experiments should include pH-buffered treatments to isolate metabolic from pH effects.

Single strain and coal type. Only *P. putida* CICC21884 was tested on lignite from one mining area. Natural microbial consortia with complementary enzymatic capabilities may exhibit synergistic effects (Huang et al., 2022b; Xue et al., 2022). Different coal ranks may respond differently to microbial treatment. Future research should evaluate additional strains (Sphingomonas, Gordonia, Rhodococcus) and establish applicability ranges across coal ranks. It should also be noted that process parameters (temperature, bacterial concentration, coal particle size) were not systematically optimized. Laboratory scale (5 g coal, 200 mL flasks) requires validation at pilot scale (100–1000 L bioreactors) and field trials on actual stockpiles with long-term monitoring (6–12 months). Post-treatment characterization should assess calorific value, grindability, and gasification reactivity to evaluate impacts on downstream coal utilization (Han et al., 2022; Zhang et al., 2024).

Mechanistic verification gaps. While temporal correlations exist between enzyme activities and functional group changes, direct causality requires: enzyme purification and *in vitro* coal treatment, specific inhibitor studies, gene knockout mutants, transcriptomics to identify upregulated genes, and metabolomics to track degradation products (Song et al., 2023; Zhao, 2022; Zhou et al., 2025).

## 5 Conclusion

This study demonstrates that *P. putida* is associated with reduced oxidative spontaneous combustion of lignite through surface-level enzymatic degradation mechanisms. Microbial treatment during the optimal 24–-72 h window elevated the ignition temperature by 23.95 °C and enhanced the thermal stability of lignite, correlating with reduced content of active functional groups, particularly hydroxyl (39%→31%) and oxygen-containing functional groups (41%→36%), alongside increased aromatization (17%→30%). Three extracellular enzymes—aromatic acid monooxygenase (22.0 U/mL), esterase (68.5 U/mL), and catalase (125.5 U/mg protein)—exhibited coordinated peak activities at 24 h, coinciding with the period of maximal functional group modifications. Linear regression analysis across seven treatment conditions revealed general trends between functional group composition and thermal stability parameters, with oxygen-containing groups showing weak positive association with critical temperature (R^2^ = 0.11), aromatic content showing weak positive trend with ignition temperature (R^2^ = 0.26), and hydroxyl content showing weak negative trend with maximum weight loss rate (R^2^ = 0.26), though substantial variability was observed due to limited sample size and outlier effects. These structural modifications were associated with 53.75% reduction in weight-loss rate and spontaneous combustion propensity index reaching 540.15. However, treatment exceeding 120 h caused functional group rebound potentially related to bacterial autolysis, underscoring the necessity for strict duration control. The findings suggest potential enzymatic mechanisms for microbial inhibition of lignite spontaneous combustion, pointing toward an environmentally benign and efficient technical approach with optimizable treatment parameters.

## Data Availability

The original contributions presented in the study are included in the article/supplementary material, further inquiries can be directed to the corresponding author.
